# Randomized Controlled Trial: Preliminary Investigation of the Impact of High-Intensity Treadmill Gait Training on Recovery Among Persons with Traumatic Brain Injury

**DOI:** 10.1089/neur.2024.0169

**Published:** 2025-01-24

**Authors:** Tyler Shick, Courtney Perkins, Arco Paul, Melissa Martinez, Joseph Joyce, Katy Beach, Jeffrey Swahlan, Justin Weppner

**Affiliations:** ^1^ Virginia Tech Carilion School of Medicine, Roanoke, Virginia, USA.; ^2^ Carilion Clinic, Roanoke, Virginia, USA.; ^3^ Radford University Carilion, Roanoke, Virginia, USA.; ^4^ Edward Via College of Osteopathic Medicine, Blacksburg, Virginia, USA.

**Keywords:** aerobic exercise, high-intensity gait training, physical therapy, traumatic brain injury

## Abstract

Exercise to treat traumatic brain injury (TBI) is a novel approach that has only become recognized in the past decade. High-intensity gait training (HIGT) has been studied in subjects following stroke; however, little research investigates similar protocols on patients with TBI. The study evaluated HIGT as an intervention for enhancing patient recovery after TBI. Adult subjects (18–65 years) who suffered TBI were randomly allocated to an intervention (HIGT) or control (low-intensity physical therapy) group given three days/week for 1 h over four weeks. Assessments included the 10-m walk test, 6-min walk test, Berg Balance Scale, five-times sit-to-stand (5TSTS), timed up and go (TUG), cognitive TUG, and Montreal Cognitive Assessment (MoCA) at day one, two weeks, four weeks, and a four-week follow-up. In addition to a trend toward improved gait speed (*p* < 0.1) and significantly improved endurance (*p* < 0.05) in the HIGT group (*n* = 5), both the control (*n* = 4) and HIGT groups demonstrated trends toward improved mobility (5TSTS, *p* < 0.1; TUG, *p* < 0.1) and significantly improved cognition (cognitive TUG, *p* < 0.01; MoCA, *p* < 0.05) over the four-week time period and at the one-month follow-up. HIGT showed longer-lasting rehabilitative effects on gait distance, endurance, mobility, and cognitive function at the four-week follow-up. This study suggests that HIGT may support functional recovery, and future work will involve increasing sample size.

## Introduction

In 2015, the Centers for Disease Control and Prevention declared traumatic brain injuries (TBIs) a “silent pandemic.” From 2002 to 2010, at least 2.5 million emergency department visits, hospitalizations, and deaths were attributed to TBI in the United States annually.^1^ Survivors often face physical disabilities, cognitive impairments, and limitations in social and leisure activities, yet optimal treatment regimens remain elusive.^2^

Exercise as a treatment for TBI has only gained recognition in the past decade, particularly for mild cases. At the turn of the millennium in 2001, the First International Conference on Concussion in Sport recommended complete brain rest after mild TBI.^3^ However, a subsequent study suggested that moderate activity, such as slow jogging and mowing the lawn, could improve neurocognitive function and symptoms after mild TBI, shifting the paradigm toward activity-based interventions.^4^ By 2012, the Fourth International Conference acknowledged the value of low-intensity exercise (i.e., walking) post-TBI.^5^ In 2019, a University at Buffalo randomized clinical trial demonstrated that subsymptom aerobic exercise shortened recovery time in adolescents with mild TBI.^6^ Despite progress with mild TBI, studies on activity-based therapy for moderate-to-severe TBI lag, where current rehabilitation often includes more intensive interventions tailored to motor and cognitive deficits. Within two decades, the standard of care, particularly for mild TBI, has shifted from complete rest to more intense exercise, yet the benefits of high-intensity exercise and activity-based therapy for moderate-to-severe TBI remain to be fully explored.

Previous research had been conducted on the efficacy of high-intensity gait training (HIGT) in subjects following stroke. Within this research, HIGT is typically defined as walking with or without an assisted device at 60–85% of heart rate reserve (HRR). Studies found that aerobic exercise at 70–85% age-predicted maximum HR significantly improved subjects’ 10-m walk test (10MWT) (self-selected and fastest possible speeds), Berg Balance Scale (BBS), and 6-min walk Test (6MWT). Additionally, high-intensity locomotor training at 70 − 80% of HRR yielded significantly better 6MWT outcomes compared to a low-intensity group at 30–40% HRR.^7,8^

Along with its use in the stroke population, aerobic exercise has already been shown to improve recovery after TBI in animal models. On a microscopic level, aerobic exercise has been shown to increase cerebral growth factors, enhance neurogenesis, improve neuron survival and regeneration, reduce lesion size, and modulate inflammatory responses. Animal studies consistently demonstrate that exercise is neuroprotective and promotes recovery. This can be observed at the macroscopic level as enhanced cognitive function, reduced depression, and decreased anxiety.^9^ For instance, a study in murine models found that 10 days of moderate intensity treadmill exercise after controlled cortical impact reduced anxiety-like behavior, improved spatial memory, and promoted hippocampal proliferation and survival.^10^ In rats, just a single session of low-intensity exercise early post-TBI improved behavioral deficits.^11^

Although stroke (metabolic stress) and TBI (mechanical damage) are two different initial insults, both lead to white matter vulnerability, resulting in functional abnormalities. The two different insults produce disturbances in ion homeostasis and cause similar neurochemical responses, leading to excitotoxicity from glutamate release and acidotoxicity due to failure of Na^+^/K^+^ pumps, ultimately resulting in neuronal cell death. Furthermore, inflammatory processes through blood leukocytes, soluble factors (i.e., reactive O_2_ and tumor necrosis factor alpha), and reactive astrocytes similarly contribute to pathogenesis of both injuries. In addition, cerebral edema due to disruption of the blood brain barrier and osmolarilty/cytotoxic changes can increase risks of elevated intracranial pressure.^12^ Therefore, we believe that HIGT can be used for TBI rehabilitation because of its similarity to stroke.

Despite the high prevalence and severe consequences of TBIs, there is still a significant lack of evidence for the best treatment strategies. This study aims to explore the potential of HIGT to better ameliorate motor and cognitive deficits post-TBI, building on evidence from stroke rehabilitation and animal models. This study evaluated the treatment strategy of HIGT post-TBI based on gait speed, endurance, balance, functional mobility, and cognitive function compared with a low-intensity physical therapy program. Based on these parameters, we hypothesized that HIGT would be a more effective treatment for TBI compared with the low-intensity physical therapy program.

## Methods

### Design and sample

This study was a randomized clinical trial (RCT) preliminary feasibility study consisting of two groups—an intervention group (HIGT) and a control group (low-intensity physical therapy without HIGT)—prescribed during the subacute to chronic phase following a mild-to-severe TBI. This trial was approved by the institutional review board committee (IRB-21-1512) and registered with ClinicalTrials.gov (NCT05622786). Written informed consent was obtained from all human subjects as required. The study was conducted at an outpatient rehabilitation facility. The clinic had treadmills for HIGT and equipment for low-intensity physical therapy.

Potential subjects were identified through medical records. These subjects were recruited from a single academic brain injury center. Interested candidates were given 24 h to return a signed consent form. Inclusion criteria involved adult subjects (18–65 years) with mild, moderate, or severe TBI (initial or recurrent), evaluated by an experienced clinician who classified the severity of the TBI. Exclusion criteria included (1) unstable orthopedic conditions, such as unstable craniectomies or weight-bearing restrictions; (2) unstable cardiac conditions, including unstable angina, unstable cardiac dysrhythmias, myocardial ischemia, or hypertension at rest (systolic BP >140 mm Hg and diastolic BP >90 mm Hg, unless cleared by a physician if above this range); (3) acute systemic infection with fever, body aches, or swollen lymph glands; (4) hospitalization for acute cardiac, pulmonary, or metabolic conditions in the past 3 months; (5) any other physical or mental restrictions preventing participation in the research protocol; and/or (6) pregnancy.

Subjects meeting study criteria were initially screened on a treadmill to assess their HIGT tolerance. The screening process monitored HR and blood pressure, while the subject underwent a single session of walking on the treadmill with gradually increasing intensity, with or without an assistive device. The speed was gradually increased to reach HR of 85% HRR, which was calculated using the formula (max HR − baseline HR) × 0.85 + baseline HR.^7,8^ Baseline HR was measured before treadmill walking. Max HR was calculated as (208 − [0.7 × age]), based on a cross-validated meta-analysis.^13^ HR data were collected using the Polar H10 HR chest strap monitoring sensor, which assessed the time elapsed between two successive R-waves of the QRS complex with high accuracy and reliability compared with clinical-grade electrocardiograms.^14^ Throughout this screening, adverse signs, such as myocardial ischemia (identified by angina, shortness of breath, nausea, vomiting), a drop in systolic BP >10 mm Hg from baseline despite increasing workload, hypertensive response (systolic BP >220 mm Hg or diastolic BP >115 mm Hg), or significant oxygen saturation changes, were monitored. If any of these signs appeared, the test was stopped, and the participant was excluded. If a subject experienced dizziness, nausea, confusion, tingling, and numbness in the hands or feet, musculoskeletal pain, or other adverse events, they were asked if they wanted to continue. If they did, their condition was documented and assessed by a clinician. If cleared, they stayed in the study; if not, they were excluded and advised to consult their physician.

Subjects were randomly assigned to either the intervention or control group by the principal investigator using Microsoft Excel’s random number generator formula. Over four weeks, they underwent either HIGT combined with low-intensity physical therapy (intervention) or low-intensity physical therapy alone (control), with 1-h sessions three days per week. This study’s schedule was based on stroke literature showing significant improvements with 12 or fewer high-intensity locomotor training sessions over four to five weeks.^8^ HIGT group participants started with a 3-min treadmill warm-up (30–50% HRR), followed by 25 min of HIGT (60–85% HRR) and ended with a 2-min cool-down (30–50% HRR). The remainder of the 60-min therapy session was allocated to low-intensity physical therapy activities, which comprised balance training, over-ground gait training with/without dual-tasking, and stretching exercises. HIGT was administered by Doctors of Physical Therapy trained to strictly follow HIGT protocol, while all low-intensity treatment plans were led by a single Doctor of Physical Therapy to ensure consistency. Standard of care at this study’s outpatient physical therapy services for TBI patients include therapeutic exercises (both aerobic and strength training types), balance training activities, stretching exercises, cognitive training, and various types of functional training activities including gait training; however, higher intensities were not emphasized.

At admission (day 1), two weeks (day 14), four weeks (day 28), and four weeks post-discharge (1 month follow-up/day 56), participants in both groups were assessed using the 10MWT for gait speed, 6MWT for gait distance and endurance, BBS for balance, five-times sit-to-stand (5TSTS) and timed up and go (TUG) for functional mobility, and cognitive TUG and Montreal Cognitive Assessment (MoCA) for cognitive function. Each measure was selected for its established reliability and relevance to the study outcomes, with detailed protocols and scoring criteria well documented in the literature.^15–19^ All assessments were conducted by trained study personnel to maintain high interrater reliability.

### Data analysis

Collected data were compiled in REDCap to ensure confidentiality. Data analysis was performed with GraphPad Prism, Version 9 for Mac, using mixed-effects analysis with the restricted maximum likelihood model to compare HIGT and control groups. Quantitative data were expressed as mean ± standard error of the mean (SEM). *p* < 0.1 represented a trend toward significance, and *p* < 0.05 was considered statistically significant. In addition, an unpaired *t-test* was used to compare the HIGT and control groups at designated time intervals.

## Results

In a four-week study with a four-week follow-up, we evaluated the effects of HIGT (*n* = 5) in comparison to low-intensity physical therapy (Control [*n* = 4]) on gait speed, endurance, functional mobility, cognitive function, and balance. Participants in the study included White males (*n* = 5) and females (*n* = 4) between ages 19 and 33 with mild (*n* = 6), moderate (*n* = 1), or severe (*n* = 2) TBI randomly allocated between the two groups. TBI injury date ranged between 9 and 1302 days before the start of the study with various etiologies, such as motor vehicle accidents and sports injuries.

Thirteen individuals met inclusion criteria and consented to participate in the study, but two did not pass the screening. One participant failed the screening due to a worsening migraine headache, while another was unable to pass due to elevated blood pressure. Out of the 11 participants who passed the screening, two withdrew from the study due to transportation issues. Hence, the study was completed by nine participants. One participant from the intervention group who completed the study was unavailable for the one-month follow-up.

There were no demographic differences in race. The variances in sex, TBI severity, and time since injury were evenly spread across both groups. The intervention group (mean age = 26.60) was slightly older than the control group (mean age = 24.75) but not statistically significant (Table 1). The timeframe of recruitment lasted from October 2022 to June 2023. There were no adverse events reported. Due to the study’s nature, participants were not blinded to their assigned study group. The analyses performed were based on the patient’s originally assigned group.

**Table 1.** Demographics (# of Adults in Each Group)

	Control (*n* = 4)	HIGT (*n* = 5)
Gender		
Male	2	3
Female	2	2
Age^a^		
18–29	4	2
30–39	0	3
TBI severity		
Mild	3	3
Moderate	0	1
Severe	1	1
Time of enrollment post-TBI		
<3 months	2	3
3–6 months	1	0
6 months–1 year	0	1
1–3 years	1	0
3–5 years	0	1

^a^ Unpaired *t-*test, *p* = 0.6316.

HIGT, high-intensity gait training; TBI, traumatic brain injury.

**FIG. 1.**
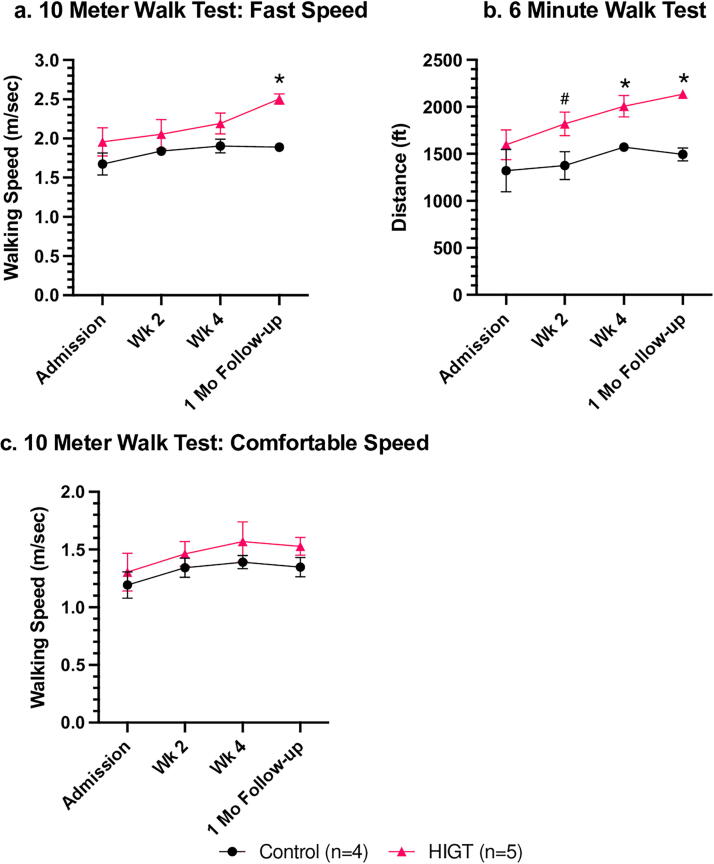
The HIGT group showed significant improvements in gait speed when walking at a fast speed and endurance compared to the baseline and the low-intensity physical therapy group. **(a)**. Mean ± SEM values for 10-m walk test at a fast speed. Mixed-effects analysis showed a significant main effect of time (*p* < 0.05) and a trend toward significance for group (*p* < 0.1). Asterisks (*p < 0.05) indicate a significant difference on unpaired *t-test*, respectively. **(b)** Mean ± SEM values for 6-min walk test. Mixed-effects analysis showed a significant main effect of time (*p* < 0.05) and group (*p* < 0.05). Asterisks (*p < 0.05) or pound sign (#*p* < 0.1) indicate a significant difference or trend toward significance on unpaired *t-test*, respectively. **(c)** Mean ± SEM values for 10-m walk test at a comfortable speed. Mixed-effects analysis showed no significant main effect of time (*p* = 0.1157) nor significant main effect of group (*p* = 0.3339). Unpaired *t*-test revealed no significant difference between groups at specific time points. HIGT, high-intensity gait training; SEM, standard error of mean.

The evolution of participants’ walking capabilities (Fig. 1) was illustrated by gait speed (10MWT) and endurance (6MWT). In the 10MWT at a fast speed and 6MWT, both groups showed significant improvement over time (*p* < 0.05), with the HIGT group trending better in the 10MWT (*p* < 0.1) and performing significantly better in the 6MWT (*p* < 0.05) compared with low-intensity physical therapy. In the 10MWT at a comfortable speed, there was no significant enhancement in gait speed throughout the study. Moreover, the HIGT group did not exhibit significantly superior gait speed when compared to the control group. Both groups showed quantitative (mean) enhancements in gait speed at a comfortable pace over time. At admission, the HIGT group started the study with slightly higher initial walking speeds and endurance measures, though these differences were not statistically significant. At week 2 (*p* < 0.1) and week 4 (*p* < 0.05), the HIGT group was able to traverse farther distances in the 6MWT compared to the control group. At follow-up, compared to the control group, the HIGT group had significantly faster mean walking speeds, along with greater retention and further improvement in endurance capacity based on distance covered during the 6MWT (*p* < 0.05). The HIGT group sustained the enhancements achieved during the study in both the 10MWT at a fast pace and the 6MWT, whereas the control group did not maintain these improvements. Some of the improvements in gait speed at a comfortable pace were also not sustained at the 1-month follow-up.

**FIG. 2.**
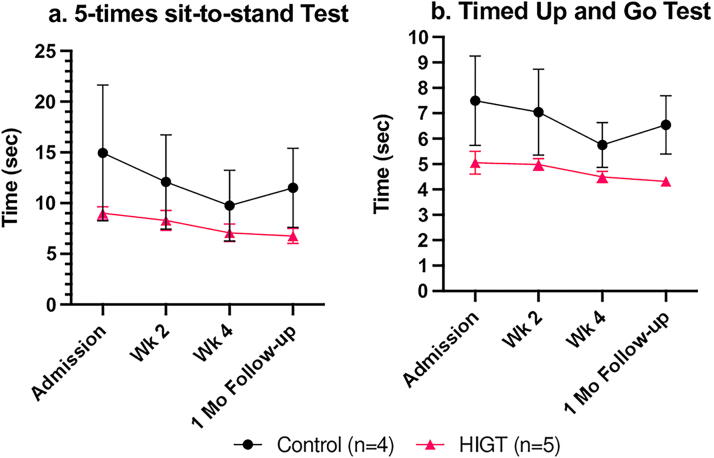
Both intervention and control groups showed improvements in mobility over time. The HIGT group, however, exhibited longer-lasting rehabilitative effects during the four-week follow-up. (a) Mean ± SEM values for five-times sit-to-stand test. Mixed-effects analysis showed a trend toward significance for time (*p* < 0.1), but no significant main effect of group. **(b)** Mean ± SEM values for timed up and go test. Mixed-effects analysis showed a trend toward significance for time (*p* < 0.1), but no significant main effect of group. HIGT, high-intensity gait training; SEM, standard error of mean.

When examining functional mobility using the 5TSTS and TUG, both groups showed a tendency to improve over time (*p* < 0.1), without any group differences on the duration taken to complete the 5TSTS and TUG tests (Fig. 2). In general, the HIGT group had faster 5TSTS and TUG times at admission, although not statistically significant. Unlike the 10MWT and 6MWT, there was no significant difference between the HIGT and control groups at any specific timepoint in the 5TSTS and TUG assessments. Nevertheless, the HIGT group quantitatively demonstrated more long-lasting effects in mobility upon assessment with the two tasks at the 1-month follow-up.

**FIG. 3.**
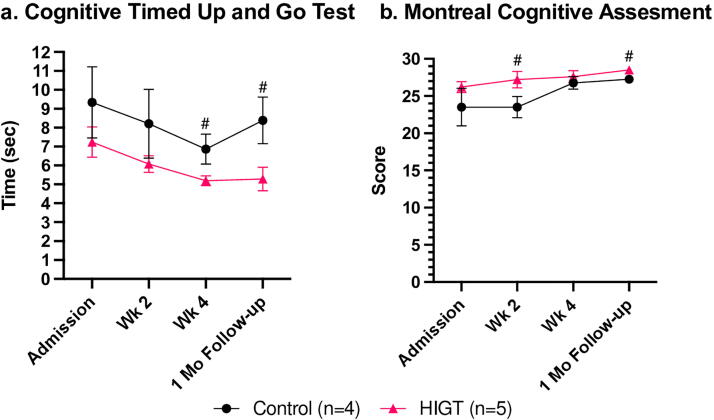
Both intervention and control groups showed improvements in cognitive function over time. The HIGT group, however, exhibited longer-lasting rehabilitative effects during the four-week follow-up. (a) Mean ± SEM values for cognitive timed up and go test. Mixed-effects analysis showed a significant main effect of time (*p* < 0.001), but no significant main effect of group. Pound sign (#*p* < 0.1) indicates a trend toward significance on unpaired *t-test*. **(b)** Mean ± SEM values for Montreal cognitive assessment test. Mixed-effects analysis showed a significant main effect of time (*p* < 0.05), but no significant main effect of group. Pound sign (#*p* < 0.1) indicates a trend toward significance on unpaired *t-test*. HIGT, high-intensity gait training; SEM, standard error of mean.

The evolution of cognitive function over time for both the HIGT and control groups was illustrated by the cognitive TUG and MoCA (Fig. 3). Both cohorts exhibited enhanced cognitive abilities since the study’s initiation, which revealed a statistically significant trend over time (*p* < 0.05) across both cognitive assessment instruments. However, there was no significant main effect of group in either assessment when comparing HIGT and control. At the beginning of the study, the HIGT group completed the cognitive TUG more quickly and achieved a higher score on the MoCA compared with the control group, although this difference was not statistically significant. At the two-week timepoint, the control group scored lower in the MoCA (*p* < 0.01), and at the four-week timepoint, the control group spent more time to complete the cognitive TUG (*p* < 0.1), representing poorer cognitive attention. Moreover, at the four-week follow-up, a trend toward significance (*p* < 0.1) was observed utilizing the unpaired *t-test* when comparing the time spent completing the cognitive TUG and MoCA scores of the HIGT and control group, with the HIGT group outperforming. Interestingly, at the four-week follow-up, HIGT appeared to exhibit longer-lasting improvements on cognitive attention compared to the control group, most evident in the cognitive TUG.

**FIG. 4.**
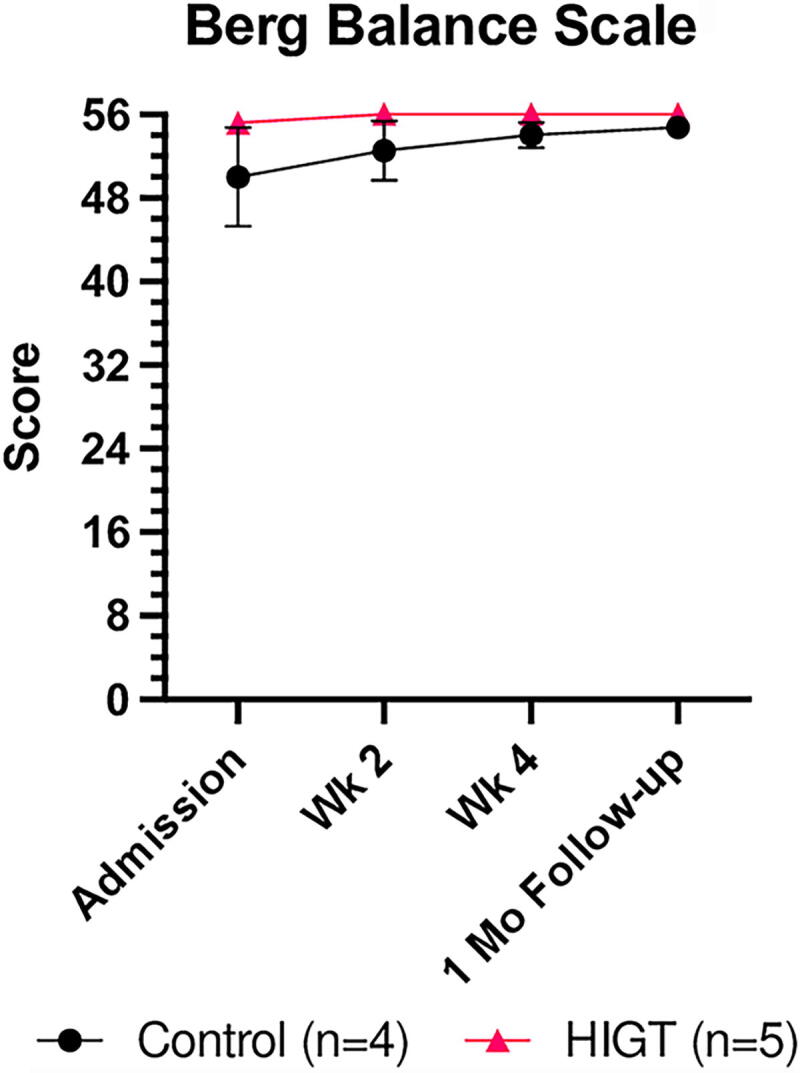
Both intervention and control groups showed quantitative improvements in balance over time. Mean ± SEM values for Berg Balance Scale. Mixed-effects analysis showed no significant main effect of time (*p* = 0.2185) nor significant main effect of group (*p* = 0.1863). Unpaired *t-test* revealed no significant difference between groups at specific time points. SEM, standard error of mean.

Although the HIGT group demonstrated enhancements in gait speed and endurance relative to the control group, there was no significant enhancement in balance throughout the study (Fig. 4). Additionally, the HIGT group did not achieve significantly better BBS scores compared to the control group. At admission, the HIGT group began with noticeably higher BBS scores, although this was not statistically significant. The control group showed quantitative improvements in balance over time, but individual scores varied widely from the mean (SEM). The HIGT group also exhibited modest improvements in balance capabilities. A ceiling effect was observed in both groups as most participants nearly maxed out their score by the end of the study. Given that a BBS of <45 indicates risk of fall, there was no risk of fall for participants in either group throughout the study.

## Discussion

In this RCT, the impact of HIGT on gait speed, endurance, functional mobility, and cognitive function was assessed in comparison to individuals undergoing low-intensity physical therapy following TBI. This is the first RCT to evaluate HIGT as a therapeutic intervention post-TBI. Over four weeks, the HIGT group showed significantly greater improvements in fast-paced walking abilities and endurance compared with the control group. At the one-month follow-up, the HIGT group also exhibited more long-lasting improvements in fast-paced gait speed, endurance, and cognitive function than those receiving low-intensity physical therapy. HIGT showed a tendency for longer-lasting mobility effects compared with the control group though not statistically significant. HIGT did not significantly impact balance capabilities compared to controls. A ceiling effect was observed in both groups, with most participants nearly reaching the maximum BBS score, making it unable to capture small, clinically meaningful gains, even if their balance continued to improve. Future studies should consider alternative balance measures like the High Level Mobility and Assessment Tool or the Community Balance and Mobility Scale. This study revealed that HIGT was both safe and well received by TBI participants who underwent thorough screening, underscoring the significance of high-intensity aerobic activity for patients with TBI and its integration into treatment plans for clinically stable individuals.

Previous research has shown the benefits of aerobic exercise following TBI. Animal studies indicate that exercise positively impacts cognitive recovery, enhancing performance, boosting neuroplasticity, and supporting neuronal repair and regeneration. Consistent with these findings, this study’s results suggest that high-intensity aerobic exercise is advantageous regardless of when treatment starts after the injury (from 9 to 1302 days post-TBI). However, there is evidence suggesting that animals beginning with immediate access to running wheels after TBI may encounter adverse effects, implying that initiating exercise during the acute phase of TBI could have unintended consequences. Moreover, delayed exercise was linked to increased cognitive performance and brain-derived neurotrophic factor levels.^20,21^ Since some preclinical studies have shown that starting very early physical activity post-TBI can worsen post-traumatic neurodegenerative processes, it remains uncertain whether HIGT would be beneficial to individuals who experienced a TBI within the first week. In the stroke population, studies have found initiation of intensive therapy within the first 24 hours to have harmful effects, suggesting this may also be the case for TBI with their similarities in pathophysiological responses.^22^ Given the complexities of human physiology compared with animal models, future research in TBI assessing this topic would be valuable for determining the optimal timing to initiate treatment.

Recent clinical and retrospective studies involving adolescents have shown that engaging in moderate-intensity exercise improves symptom scores and accelerates recovery following mild TBI.^4,6^ These studies have limitations, however, as they exclusively focused on individuals under 18 years old and specifically included only those with mild TBIs. Furthermore, they emphasized intensity level as an important factor to consider when initiating an aerobic exercise program. Other studies with animals have also highlighted the significance of intensity level in utilizing exercise as a cognitive rehabilitation method, where low and moderate intensity were found to yield the most significant benefits. In these studies, high intensity resulted in nonsignificant improvements, although there remains varying consensus on what intensity is most effective.^9,23–25^ In contrast to existing literature, this study indicates potential for high-intensity interventions in the TBI population. Furthermore, HIGT seemed advantageous for individuals across all TBI severity levels, ranging from mild to severe. Supporting these findings, a recent pilot randomized controlled trial demonstrated that high-intensity stepping significantly improved 6MWT performance and peak treadmill speed in individuals with chronic TBI.^26^ Although high-intensity exercise is a novel approach for TBI rehabilitation, HIGT has been extensively studied in the stroke population, showcasing improvements in gait speed, balance, and endurance.^7,8^ Due to the similarities between strokes and TBI, it is understandable to observe improvements in the TBI population with HIGT.

High-intensity aerobic exercise has been demonstrated to increase various blood biomarkers (including s100 calcium-binding protein beta, neuron-specific enolase, brain-derived neurotrophic factor, neurogranin, peroxiredoxin-6, creatinine kinase-BB isoenzyme, von Willebrand factor, monocyte chemoattractant protein-1, matrix metalloproteinase-9, and total tau) after a single session in healthy males. Interestingly, after six sessions, total tau returned to baseline, and there was significant attenuation in neurogranin, peroxiredoxin-6, matrix metalloproteinase-9, and von Willebrand factor levels.^27^ This suggests high-intensity exercise may play a role in regulating brain-derived biomarkers after injury, although the mechanism for influencing recovery after a TBI remains largely unknown. Another study with post-stroke patients discovered that high-intensity interval training led to acute elevations in circulating brain-derived neurotrophic factor and corticospinal excitability compared to those engaged in moderate-intensity continuous exercise.^28^ Therefore, high-intensity exercise may have the potential to improve the brain’s receptiveness to rehabilitation through the elevation of brain-derived neurotrophic factor levels and corticospinal excitability.

This study is the first RCT to evaluate HIGT as a therapeutic intervention for TBI, demonstrating significant improvements in gait speed, endurance, and cognitive function compared with low-intensity physical therapy. However, there are some limitations to consider. First, this study did not address the mechanisms underlying how exercise might affect recovery following TBI. Additionally, the limited sample size in this feasibility study could have influenced this study’s findings. While not statistically significant, the HIGT group began with better assessment scores, indicating that this group might have been higher functioning at the beginning of the study. This small sample size also led to high standard error. Additionally, there is a possibility that participants engaged in aerobic exercise outside of the study without the researchers’ knowledge, potentially influencing their recovery outcomes. This study also only enrolled adults between the ages of 19 and 33, so the results cannot necessarily be generalized to younger children or older adults.

This feasibility study provides initial insights, but larger studies are necessary to confirm and expand upon its findings. Future research should employ larger sample sizes to enhance the robustness and generalizability of the results to the broader population. By increasing the sample size, future studies can provide more comprehensive evidence on the effectiveness of HIGT for individuals with TBI. Future RCTs should also investigate the effectiveness of HIGT compared with moderate-intensity exercise and elucidate the mechanisms that underlie the benefits of HIGT.

## Conclusion

In this feasibility study, we demonstrated the advantages of HIGT as a therapeutic intervention after TBI. With future research endeavors involving larger sample sizes and a more diverse population, it will be possible to gain a deeper understanding of HIGT’s role in TBI recovery. The findings in this study suggest that HIGT may be a safe and promising intervention for addressing TBI, potentially integrating into comprehensive rehabilitation strategies to enhance patient outcomes and quality of life.

## Transparency, Rigor, and Reproducibility Summary

The study was approved by the Institutional Review Board (IRB-21-1512) and registered on ClinicalTrials.gov (NCT05622786), ensuring transparency and adherence to ethical standards. Written informed consent was obtained from all participants prior to enrollment, following ethical guidelines. Detailed inclusion and exclusion criteria ensured that the study targeted individuals with a range of TBI severities while excluding those with unstable medical conditions or other physical limitations that could interfere with the intervention. The study recruited 13 participants, with 11 passing the initial screening. Two were excluded due to adverse responses (worsening migraine and elevated blood pressure). Nine participants completed the study: five in the HIGT group and four in the control group. Participants were White males (*n* = 5) and females (*n* = 4), aged 19–33 years, with mild (*n* = 6), moderate (*n* = 1), and severe (*n* = 2) TBI, caused by motor vehicle accidents or sports injuries. Time since injury ranged from 9 to 1,302 days. Two participants withdrew due to transportation issues, and one in the intervention group was unavailable for the one-month follow-up. Recruitment occurred from October 2022 to June 2023. The data were managed securely using REDCap, with strict measures to ensure participant confidentiality. To enhance reproducibility, this study utilized validated equipment and standardized protocols for both intervention and control groups. A detailed description of the HIGT and control interventions is provided to facilitate replication in future studies. Data collection methods and statistical analyses are clearly defined, supporting both internal and external validity. Furthermore, the use of widely accepted functional and cognitive assessments, along with a rigorous randomization process, underscores the study’s commitment to scientific rigor. No blinding was applied due to the study’s nature. Data analysis was conducted using GraphPad Prism, employing mixed-effects analysis and unpaired *t*-tests to compare outcomes between the HIGT and control groups. The datasets generated and analyzed in the current study are not publicly available. However, this information is available from the corresponding author upon request.

## Abbreviations Used

TBI: Traumatic Brain Injury
TBIs: Traumatic Brain Injuries
HIGT: High-intensity gait training
HRR: Heart rate reserve
HR: Heart rate
5TSTS: Five-times sit-to-stand
TUG: Timed up and go
10MWT: 10-m walk test
BBS: Berg Balance Scale
6MWT: 6-min walk Test
RCT: Randomized clinical trial
MoCA: Montreal Cognitive Assessment
